# Bispecific immune molecule PD1-IL2v: a new therapeutic strategy for pancreatic ductal adenocarcinoma

**DOI:** 10.1038/s41392-023-01611-4

**Published:** 2023-10-06

**Authors:** Yuan Gao, Liqing Yu, Hongming Miao

**Affiliations:** 1 Department of Pathophysiology, College of High Altitude Military Medicine, Army Medical University, Chongqing, 400038 China; 2 Division of Endocrinology, Diabetes, and Nutrition, Department of Medicine, University of Maryland School of Medicine, Baltimore, MD USA; 3 Jinfeng Laboratory, Chongqing, 401329 China

**Keywords:** Cancer microenvironment, Tumour immunology, Immunotherapy

Recently, Piper and Hoen et al. reported a new immunotherapy for pancreatic cancer in orthotopic pancreatic ductal adenocarcinoma (PDAC) KPC tumor model.^1^ They found that a murine PD-1-targeted IL-2 variant complex (PD1-IL2v), in combination with radiation therapy (RT), can inhibit PDAC growth and metastasis by immune system, and induce a lasting immunological memory response to the tumor.

Pancreatic cancer is one of the malignant tumors with the highest mortality rate in the world.^2^ Due to the low immunogenicity of PDAC, all immunotherapy has not yet been able to provide results that can improve survival rates like other solid tumors.^2^ The role of radiotherapy in the treatment of pancreatic cancer has also been questioned.^3^ Interestingly, a phase III clinical trial of a combination therapy method of PDAC also based on IL-2v and PD-L1 failed earlier. But the therapy used in the clinical experiment is actually different from the one used in this article.

Interleukin 2 (IL-2), also known as T cell growth factor, is mainly produced by activated CD4^+^ T cells and CD8^+^ T cells.^4^ It is a growth factor for all T cell subpopulations, though also acting on IL-2 receptor-expressing NK cells^5^ et al. In addition, IL-2 can interact with IL-2Rα with high affinity, promoting the maintenance and expansion of Treg cells.^4^ PD-1 is an immunosuppressive factor targeted in current immunotherapy, but its efficacy in PDAC is limited. While activation of IL-2 signaling may help improve cancer immunotherapy, its high affinity with IL-2Rα may lead to vascular leak syndrome and needs to be inhibited. Piper and Hoen et al.^1^ utilized a high-affinity anti-PD-1 antibody fused to an IL-2 variant (IL2v) that no longer binds IL-2Rα while still stimulating tumor antigen-specific T cell expansion and differentiation to “better effectors”.

**Fig. 1:**
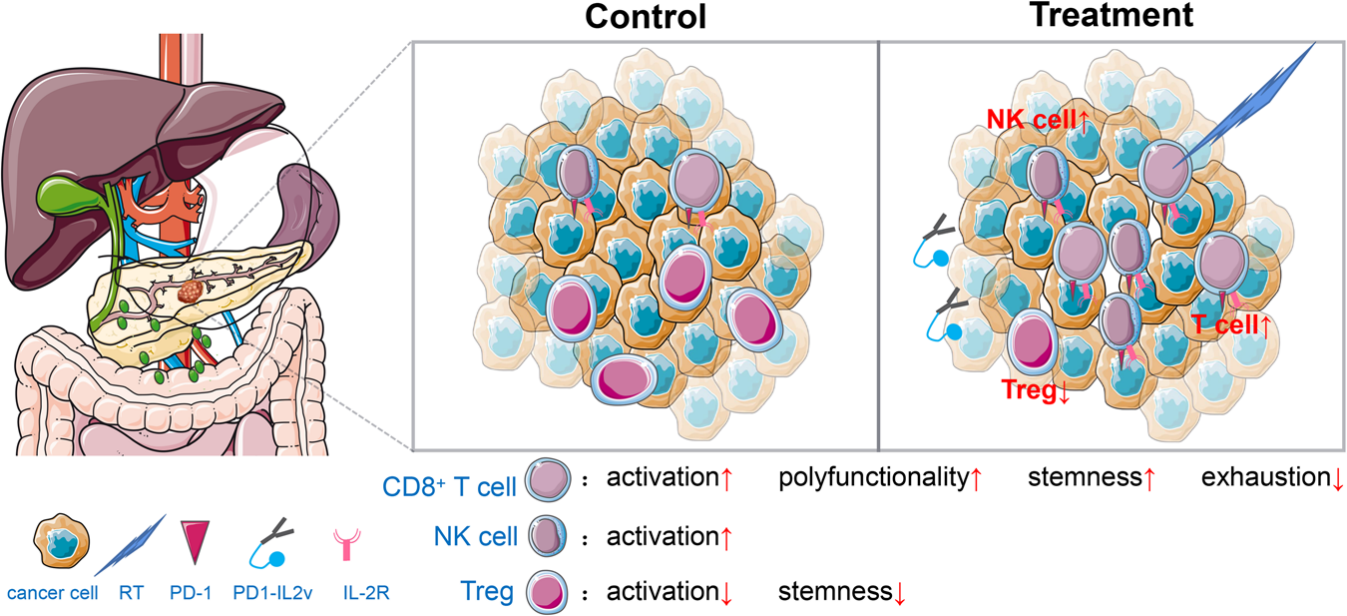
Schematic diagram of PD1-IL2v+RT inhibiting the growth of PDAC. PD1-IL2v+RT can inhibit the progression of PDAC by enhancing the activation, proliferation, invasion and migration of CD8^+^ T cells and NK cells in blood, lymph nodes and tumors, reducing the number of Treg cells and exhausting the expression of T cell-related proteins

To study the effect of this protein complex on the growth of pancreatic cancer, the authors established a mouse model of pancreatic cancer with PK5L1940 and FC1242 cell lines. They treated these animals with PD1-IL-2v, radiation therapy (RT) and other control molecules. The results showed that PD1-IL2v relative to other treatments significantly improved the survival rate of the tumor model, which was further enhanced by RT and dependent on the bispecificity of the molecule. Flow cytometry analysis showed that the number of CD8^+^ T cells increased whereas that of Treg cells decreased significantly, resulting in a significant increase in the ratio of CD8^+^ T/Treg in the PD1-IL2v-treated tumors and lymph nodes, regardless of RT therapy. The expression of proteins related to CD8^+^ T cell proliferation, function, activation, and memory also increased whereas that of CD8^+^ T cell depletion-related proteins decreased after PD1-IL2v treatment. In addition, the activation of NK cells was enhanced. Through depletion of NK and CD8^+^ T cells, they also demonstrated that these two types of cells were necessary for treatment efficacy and complemented each other’s roles. Using an OT-1 PDAC mouse model, they further established the specific role of cytotoxic T lymphocytes. Similar results were obtained from the analysis of immune cells in the blood of tumor bearing mice after PD1-IL2v treatment. These results suggested that the treatment may inhibit tumor metastasis, and indeed this was the case in a liver metastasis model. Among treatment groups, RT + PD1-IL2v and RT + PD1-IL2v + aCD25 had 1/8 and 2/8 complete response (i.e., complete clearing of tumor cells), respectively. These fully responsive animals were able to clear tumor cells again after reinjection of tumor cells on the other side due to a persistent T cell-mediated anti-tumor memory response (Fig. 1).

To explore the mechanism underlying PD1-IL2v-induced changes in CD8^+^ T cells, the authors performed the proteomic and metabolomic analysis of CD8^+^ T cells isolated from the animals. They found that protein translation and metabolic activity in these cells were significantly enhanced, and the cells were transformed into a phenotype with stronger cytotoxicity in the PD1-IL2v groups than controls.

To demonstrate clinical relevance of their findings in animal models, the authors sequenced RNAs isolated from tumor tissue samples from PDAC patients before and after clinical RT. They showed that the expression of PD-1 and PD-L1 in tumors was significantly increased after RT, with the high affinity IL-2Rα upregulated and the moderate affinity IL-2Rβ downregulated. When compared with non-responders, the responders showed decreased IL-2Rα and increased IL-2Rβ and IL-2γ. Gene set enrichment analysis found that many of the top upregulated pathways in the responders were related to immune activity with IL-2Rβ being one of frequent appearing genes. These data provide a basis for the clinical application of PD1-IL2v.

In summary, the authors introduced a complex protein molecule (PD1-IL2v) with bispecificities to PD-1 and IL-2βγ for the treatment of pancreatic cancer. The efficacy of this treatment can be enhanced by RT. PD1-IL2v treatment promotes proliferation, migration, activation, and function of CD8^+^ T cells (especially antigen specific CD8^+^ T cells) in tumors, lymph nodes, and blood. It also augments NK cell activation, reduces the expression of T cell depletion-related proteins, and suppresses proliferation and activation of Treg cells. Intriguingly, PD1-IL2v can induce the body to produce a long-lasting T cell-mediated anti-tumor memory response. The changes in CD8^+^ T cells may be caused by increased protein translation and metabolic activity. The findings are consistent with that clinical outcomes of immunotherapy for pancreatic cancer are dependent on the cell changes behind drug resistance post RT and provide a new strategy for development of immunotherapy for pancreatic cancer.
